# Effect of Prior Health Knowledge on the Usability of Two Home Medical Devices: Usability Study

**DOI:** 10.2196/17983

**Published:** 2020-09-21

**Authors:** Noémie Chaniaud, Natacha Métayer, Olga Megalakaki, Emilie Loup-Escande

**Affiliations:** 1 Centre de Rercherche en Psychologie: Cognition Psychisme et Organisations Université Picardie Jules Verne Amiens France

**Keywords:** usability, prior health knowledge, mHealth, home medical devices, blood pressure monitor, pulse oximeter

## Abstract

**Background:**

Studies on the usability of health care devices are becoming more common, although usability standards are not necessarily specified and followed. Yet, there is little knowledge about the impact of the context of use on the usability outcome. It is specified in the usability standard (ISO 9241-11, 2018) of a device that it may be affected by its context of use and especially by the characteristics of its users. Among these, prior health knowledge (ie, knowledge about human body functioning) is crucial. However, no study has shown that prior health knowledge influences the usability of medical devices.

**Objective:**

Our study aimed to fill this gap by analyzing the relationship between the usability of two home medical devices (soon to be used in the context of ambulatory surgery) and prior health knowledge through an experimental approach.

**Methods:**

For assessing the usability of two home medical devices (blood pressure monitor and pulse oximeter), user tests were conducted among 149 students. A mixed-methods approach (subjective vs objective) using a variety of standard instruments was adopted (direct observation, video analysis, and questionnaires). Participants completed a questionnaire to show the extent of their previous health knowledge and then operated both devices randomly. Efficiency (ie, handling time) and effectiveness (ie, number of handling errors) measures were collected by video analysis. Satisfaction measures were collected by a questionnaire (system usability scale [SUS]). The qualitative observational data were coded using inductive analysis by two independent researchers specialized in cognitive psychology and cognitive ergonomics. Correlational analyses and clusters were performed to test how usability relates to sociodemographic characteristics and prior health knowledge.

**Results:**

The results indicated a lack of usability for both devices. Regarding the blood pressure monitor (137 participants), users made approximately 0.77 errors (SD 1.49), and the mean SUS score was 72.4 (SD 21.07), which is considered “satisfactory.” The pulse oximeter (147 participants) appeared easier to use, but participants made more errors (mean 0.99, SD 0.92), and the mean SUS score was 71.52 (SD 17.29), which is considered “satisfactory.” The results showed a low negative and significant correlation only between the effectiveness of the two devices and previous knowledge (blood pressure monitor: *r*=−0.191, *P*=.03; pulse oximeter: *r*=−0.263, *P*=.001). More subtly, we experimentally identified the existence of a threshold level (χ²_2,146_=10.9, *P*=.004) for health knowledge to correctly use the pulse oximeter, but this was missing for the blood pressure monitor.

**Conclusions:**

This study has the following two contributions: (1) a theoretical interest highlighting the importance of user characteristics including prior health knowledge on usability outcomes and (2) an applied interest to provide recommendations to designers and medical staff.

## Introduction

### Background

Home medical devices (HMDs) are increasingly being prescribed by health care professionals in order to decongest hospitals, and they are potentially cost-effective approaches of addressing the increasing health care needs [1,2]. HMDs for the general public need to be appropriate for all types of populations regardless of the environment in which they are used [3,4]. Some of them can be very complex to use [5]. However, if HMDs are poorly designed, there could be user errors that could seriously affect patients’ safety [3,6-10].

For this reason, a number of ongoing projects are concerned with evaluating and improving the design of these medical devices [10]. User-centered methodologies [11-13] are being increasingly used as new quality and safety standards emerge [14], as a way of avoiding design errors. The “European Conformity” (CE) marking is used to prove safety and usability [15], including *effectiveness*, *efficiency*, and *satisfaction* in the specific context of use of the device. Despite all these standards, usability problems persist. Systematic analyses over the past 10 years have consistently raised an alarm [16-21] by reporting a lack of a framework and a standardized method in usability studies. Although the definition of usability is still an important debate [22], the usability standard has been updated [15] to emphasize that usability is a result of interaction rather than a property of a product [23], which is also defined by its context of use [15], and it includes the following four components: *goals* and *tasks*, *resources*, *environment*, and *users*. These components influence usability results (composed by *effectiveness*, *efficiency*, and *satisfaction*), and it is therefore necessary to know how these components specifically influence usability results. In this study, we focused on *users’* characteristics (by controlling the three other components) according to the metric of usability of the ISO 9241-11 standard [15].

Several researchers have recently investigated the links between users’ characteristics and the usability of connected devices in the health field [24-29]. The following four user characteristics seem to be particularly studied in the scientific literature:

- Age: young users outperform older users [3,4,24,25,29-32]
- Experience in information technology (IT) (or technophilia), that is, previous experience in computer and medical devices: technology experts outperform novices [3,24,32,33]
- Motivation: more motivated users outperform less motivated users [34-36]
- Health literacy: users with high levels of health literacy outperform users with low levels of health literacy [29,31,37]

With regard to the relationships between these above-mentioned characteristics and usability, there are only few studies examining the link between health [29,31,37,38] or eHealth literacy [29], particularly prior health knowledge, and usability.

The aim of this study was, consequently, to examine the effects of prior health knowledge on the usability of HMDs. In a within-subjects study design, each participant used a blood pressure monitor and a pulse oximeter.

### Related Work

#### Context of the Study

The study described in this paper is part of the *Smart Angel* project, which aims to provide individual home monitoring for patients who have undergone ambulatory surgery. This monitoring is performed for 1 week, allowing the patient to maintain connectivity with the hospital from home. The device is referred to as an eHealth system. eHealth (or connected health) is defined by Eysenbach [39] as an emerging field bringing together different disciplines such as medical informatics, public health, and business. eHealth offers an important opportunity to address the shortcomings of current health systems and to support health professionals and patients by making them actors in their own health [39,40].

This *Smart Angel* kit is composed of monitoring devices (blood pressure monitor and pulse oximeter) with a digital display application on a touch pad. These two devices were chosen by the medical collaborators of the *Smart Angel* project. They made these choices on the basis of benchmarks by selecting devices with European certification and devices considered easy to use by medical professionals. There is no link between our laboratory and the company manufacturing the equipment. Future patients will find themselves in a postsurgical context (ie, potentially with pain and nausea) at home with the devices and will have to use them (alone or accompanied) three times a day to provide updated health readings to the hospital. User needs data collected from the field in ambulatory surgery have resulted in the selection of connected devices that will monitor patients (blood pressure monitor and pulse oximeter) and that are available on the public market and carry the CE marking.

#### Usability Assessment

According to ISO 9241-11 [15], usability is defined as “the extent to which a product can be used by specified users to achieve specified goals with effectiveness, efficiency, and satisfaction in a specified context of use.” This framework makes it possible to stabilize usability around three main dimensions (*effectiveness*, *efficiency*, and *satisfaction*) that are widely used in the field of eHealth [17,21,24,41,42]. Some authors support these standards [43] and the importance of evaluating usability metrics by these three components in independent ways, as well as the collection of both subjective and objective data [17,24,44].

According to the ISO standard [15], effectiveness is defined as the accuracy and completeness with which users achieve specified goals. It is generally measured in terms of the following three points: (1) errors or difficulties in use; (2) unnecessary output elements that interfere with the user’s task; and (3) inappropriate decisions made on the basis of inaccurate or incomplete output data. According to the ISO standard [15], efficiency is defined as the resources used in relation to the results achieved (typical resources include time, human effort, cost, and materials). Efficiency includes “the time used” (ie, the time spent trying to achieve an objective).

Satisfaction is defined as the extent to which the user’s physical, cognitive, and emotional responses that result from the use of a system, product, or service meet the user’s needs and expectations. Satisfaction is assessed by physical (feelings of comfort or discomfort) and cognitive (attitudes, preferences, and perceptions) reactions [15].

#### Usability and User Characteristics

A better understanding of what influences usability results is of crucial importance to improve the design of medical devices. An increasing number of researchers are taking into account the demographic characteristics of the users they survey in their usability studies, such as age, gender, education level, IT experience, and type of disease [32,45-47]. However, very few collect user skills such as health literacy and device knowledge [29,48]. According to Borsci et al [22] and Grebin et al [49], the lack of attention to human factors is one of the reasons for the slow adoption of medical innovations. These authors proposed to better understand the factors that influence these decision-making processes in order to better understand the resilience abilities of individuals.

**Figure 1:**
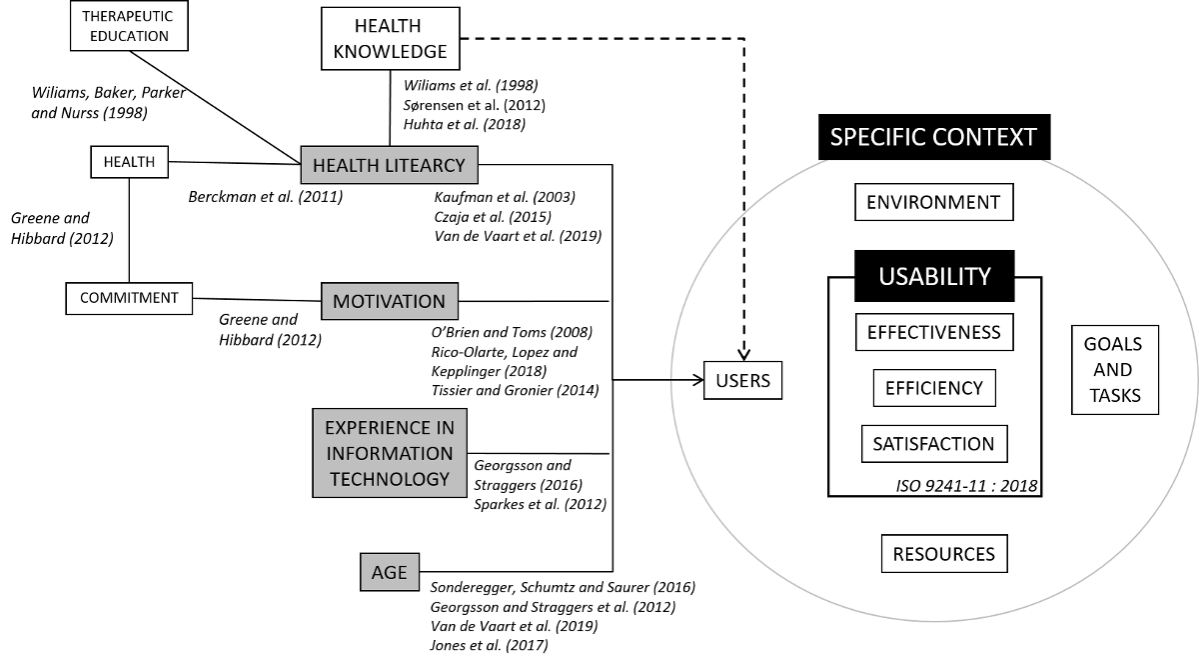
The influences of the four main user characteristics filled in grey (age, experience in information technology, motivation, and health literacy) from the scientific literature on usability results (effectiveness, efficiency, and satisfaction), which have an impact on patients’ health. Solid lines are inferred from published literature, and dashed lines are hypothetical. The solid curved line is the ISO 9241-11:2018 metric.

We were able to identify, from scientific literature, four main variables of user characteristics that directly influence the usability outcome in a “healthy” population. We propose below a nonexhaustive analysis focusing on user characteristics that influence usability results (Figure 1). These four main variables are age [3,4,24,25,29-32], experience in IT [3,24,32,33], health literacy [29,31,37,38], and motivation [34-36]. For instance, a study by Loorbach et al [50] showed that more motivating manual instructions improved effectiveness and efficiency (but not satisfaction) in relation to a mobile phone in an elderly (age 60-70 years) population. Motivation is directly linked to commitment (activation), which is also directly linked to the patient’s health [51]. In addition, age [3,25,29,32] and experience in IT [24,33] impact usability. In contrast, the influences of educational level and professional situations remain ambivalent [24,26,29].

#### Usability, Health Literacy, and Prior Health Knowledge

Health literacy seems to be an area on which many researchers are focusing to improve the design of medical devices [27,37,38,52] and thus improve the health of patients [42]. Czaja et al [37] showed that people with a low level of health literacy had difficulty completing electronic personal health records. Mackert et al [53] found that patients with low literacy levels use less health IT. Kim and Xie [38] conducted a systematic review of the impact of health literacy on health technologies. After an analysis of 74 studies, the authors concluded that the major barrier to access and use online health information for individuals with low literacy levels is strongly related to usability. A low level of health literacy can limit patients’ access to therapeutic education [54,55] and consequently lessen their commitment [56]. Paasche-Orlow and Wolf [57] even showed that literacy is directly related to health outcomes.

Even with a high level of basic literacy, a person may have difficulty obtaining, understanding, and using health information [58]. Health literacy is especially complex to assess, as studies have shown that it is not related to the age, revenue, or education of patients [59], and there is still no consensus on its definition [60]. However, most of the definitions include prior health knowledge in the concept of health literacy [60,61]. Williams et al [55] demonstrated the link between knowledge and health literacy through experimentation with patients having chronic diseases. It would appear that it is necessary to use health knowledge about how the body and treatments work in order to use health information. Thus, it is possible to suggest that better knowledge of health could lead to better usability of medical equipment.

Knowledge is often defined as a belief that is true and justified. A correct or incorrect answer is interpreted as simply meaning that a person knows or does not know something. It is necessary to integrate the “test taker’s certainty” [62] into the test in order to take into account all dimensions of knowledge. We are seeking to specifically measure knowledge about the human body in relation to the medical devices chosen, that is, to understand the functioning of arterial pressure in relation to the use of a blood pressure monitor and to understand the functioning of blood oxygenation in relation to the use of a pulse oximeter.

### Aim and Hypotheses

As we mentioned previously, theoretical studies are needed to better understand how users’ characteristics influence usability in order to design safer devices for patients. The influences of age, experience, literacy levels, and motivation on usability results have been proven (Figure 1). To our knowledge, no study has highlighted the empirical link between health knowledge and usability results. Thus, we propose to analyze this relationship by controlling the three other variables (ie, we conducted a study in a controlled environment, and we selected a young, healthy, and technologically familiar population) to limit the impact on usability results.

The aim of our experimental study was to examine the relationship between prior health knowledge and usability of HMDs. We hypothesize that participants with good prior health knowledge will use these devices with better *effectiveness* (H1), *efficiency* (H2), and *satisfaction* (H3).

## Methods

### Recruitment

One hundred and fifty-three psychology undergraduate students (mean age 20.72 years, SD 1.65 years; age range 18-32 years; 41 male and 112 female students) at the University of Picardie Jules Verne in Amiens (France) participated in the experiment. They were recruited in the university hall in March and April 2018 and were informed that they were testing medical equipment. Four participants were removed from the analysis because of technical problems. All participants were native French speakers and signed an informed consent form. The data collected on participants were anonymous. This research complied with the American Psychological Association Code of Ethics. Full review and approval were not required according to our institution’s guidelines and national regulations. Participants did not receive any financial compensation for their participation. We chose this population to avoid age bias [25] and to have homogeneous abilities in IT/computer knowledge [24]. In addition, this type of population has very little experience in the use of medical devices, making it possible to stabilize our results and avoid population-related biases.

### Materials and Measures

#### Medical Devices and Tasks

**Figure 2:**
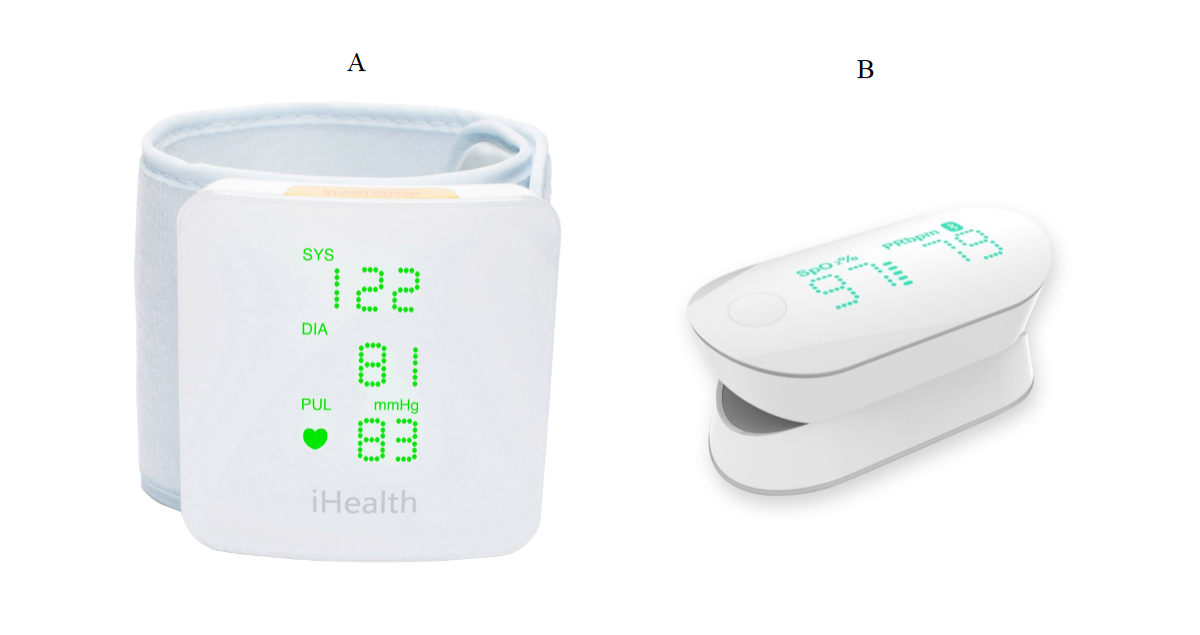
The devices used. (A) The wireless blood pressure wrist monitor (iHealth BP7). (B) The pulse oximeter (iHealth Oximeter PO3).

Participants used the following two medical devices intended for use by the general public (Figure 2): a wireless pulse oximeter (iHealth Oximeter PO3) and a wireless wrist monitor measuring blood pressure (iHealth BP7). The blood pressure monitor, once the measurement is complete, displays the systolic (*SYS*) and diastolic pressure (*DIA*) in mmHg, as well as the pulse rate next to a heart-shaped pictogram with the label *PUL*. The pulse oximeter indicates the oxygen level in the blood in %SpO_2_, as well as the pulse rate in PR bpm. The measurements and indicators (in French; eg, bpm) of each device remain displayed for a few seconds.

Ten tasks for the use of the blood pressure monitor and nine tasks for the use of the pulse oximeter were involved (Table 1).

**Table 1:** User tasks for the blood pressure monitor and pulse oximeter.

Task	Blood pressure monitor	Pulse oximeter
Task 1	Turn on the device	Turn on the device
Task 2a	Position the device correctly	Position the device correctly
Task 2b	Tilt the forearm using a system of illuminated arrows to help find the correct elbow angle to start the measurement	N/A^a^
Task 3	Start the measurement	Start the measurement
Task 4	Remain still during the measurement	Remain still during the measurement
Task 5	Record the measurement on a sheet	Record the measurement on a sheet
Task 6	Interpret the symbols on the device (eg, SYS for systolic, DYA for diastolic, and bpm for beats per minute)	Interpret the symbols on the device (eg, SPO2 for oxygen level and bpm for beats per minute)
Task 7	Interpret the results	Interpret the results
Task 8	Remove the device	Remove the device
Task 9	Turn off the device	Turn off the device

^a^N/A: not applicable.

#### Questionnaires

Participants were asked to answer the following questionnaires before and after accomplishing the task:

- Health knowledge questionnaire (before the task):
  For prior evaluation, we used a questionnaire designed in collaboration with medical and education professionals (Multimedia Appendix 1). Participants were asked to mark a number of statements as true, false, or I don’t know (eg, The heart acts like a pump). We propose a translated version (Multimedia Appendix 2) of the questionnaire in English, which requires validation.
- Personal information/use of technology questionnaire (before the task):
  This questionnaire comprised three personal details (ie, age, gender, and education level) and two items (adapted from Agarwal and Prasad [63]) related to the participant’s use of and willingness to explore innovations in IT (eg, Which of these technologies do you use and how often?). On a five-point Likert scale, the possible answers ranged from never to very often.
- System usability scale (SUS) (after the task):
  This 10-item survey aimed at recording subjective assessments of usability [64,65] is a “quick and dirty” tool with five response options from strongly agree to strongly disagree. We used the modified version described previously [66]. This version was further modified by changing the word “system” to “medical device.” This type of change has no impact on the validity or reliability of the survey instrument [67].

### Procedure

For participants, the session was divided into two parts. Each part took approximately 15 minutes. In the first part, participants completed the *personal information/use of technology* questionnaire followed by the *health knowledge* questionnaire (Multimedia Appendix 1). Participants were allowed to rest for the blood pressure measurement that followed. In the second part, they were invited by experimenters to manipulate two HMDs (oximeter and blood pressure monitor) in a randomized order. The only instruction given to the participants was to perform measurements on themselves and to record the results on their handover sheet. The participants were filmed during the session. The experiment was conducted with one participant per session in a quiet room with minimal distraction. Participants did not receive any previous training or demo from the experimenter. The experimenter only intervened when there were technical problems (eg, a battery problem). At the end of each manipulation, participants answered questions about their measurements, including questions about how to interpret the data, and they completed the SUS questionnaire (see above).

### Scoring and Statistical Analysis

The recordings were analyzed by two expert evaluators using BORIS (Behavioral Observation Research Interactive Software) [68]. ISO 9241-11 measures of effectiveness, efficiency, and satisfaction were assessed as presented below.

#### Objective Measures of Usability

In order to measure effectiveness (ie, number of handling errors) and efficiency (ie, handling time), we used the same metrics as those in the studies by Georgsson and Staggers [24] and Sheehan and Lucero [45].

The measure of effectiveness was analyzed by rating the number of handling errors, such as not putting the monitoring cuff on in the right position. Four main errors were detected for the blood pressure monitor and three for the oximeter (for instance, the participant did not hang the monitor in the right position). A scoring grid was used to identify handling errors. Participants sometimes repeated the same error several times, and we recorded the cumulative number of handling errors. The number of handling errors was between 0 and 4, except when participants repeated the same error successively. All errors were then averaged out.

The measure of efficiency was analyzed by the handling time (in seconds). The handling time was measured from the time participants first touched the device to the time they turned it off after taking the measurement.

#### Subjective Measures of Usability

In order to measure satisfaction, we used the SUS, as in other studies [24,26,69]. Scores were calculated according to Brooke guidelines [64]. The SUS score ranges from 0 to 100, with lower scores indicating lower usability.

#### Measuring Prior Health Knowledge

The *health knowledge* questionnaire (Multimedia Appendix 1) included 40 items and was divided into two parts (20 items concerning blood pressure and 20 concerning blood oxygenation). In this questionnaire, each correct answer corresponded to 1 point and each incorrect answer corresponded to 0 points, with a total score between 0 and 40.

#### Health Data Recorded

We considered whether participants were able to correctly record their blood pressure and the rate of oxygenation and whether they were able to annotate the first two measurements indicating their blood pressure and pulse oximeter. The health data recorded by the HMDs are not explicitly given. For example, participants should read three measurements on the blood pressure monitor (the first two are systolic and diastolic blood pressures and the third is pulse). Each correct measure recorded (eg, blood pressure and oxygenation rate) corresponded to 1 point, and each incorrect measure recorded corresponded to 0 points, with a total score between 0 and 1 for the blood pressure monitor and between 0 and 1 for the pulse oximeter.

#### Data Analysis

Results were computed using SPSS version 22 (IBM Corporation). Descriptive results were compared with participants’ health knowledge. For comparisons among user characteristics, user performance, and satisfaction, we compared gender, age, and IT/computer knowledge and experience (based on rated experience vs rated inexperience) against effectiveness, efficiency, and SUS mean scores for the blood pressure monitor and for the pulse oximeter. The sample did not follow normal distribution; therefore, the correlation coefficient on ranks (Spearman ρ) was used between ISO 9241-11 metrics and health knowledge. We then established three knowledge clusters (low, medium, and high levels of health knowledge) using *k*-means. The Kruskal-Wallis nonparametric test was used to analyze these clusters.

## Results

### Interjudge Reliability

We used the intraclass correlation coefficient (ICC) to verify interjudge reliability for quantitative data [70,71]. Double coding was performed on 33% of the video data collected by two independent researchers specialized in cognitive psychology and cognitive ergonomics. The average ICC measure for blood pressure monitor errors was 0.962 (95% CI 0.933-0.979; *F*_47,47_=26.461; *P*<.001). The average ICC measure for the handling time of the blood pressure monitor was 0.995 (95% CI 0.992-0.997; *F*_47,47_=261.275; *P*<.001). The average ICC measure for pulse oximeter errors was 0.936 (95% CI 0.887-0.964; *F*_47,47_=15.732; *P*<.001). The average ICC measure for the handling time of the pulse oximeter was 0.995 (95% CI 0.992-0.997; *F*_47,47_=261.275; *P*<.001).

### Internal Consistency of the Prior Health Questionnaire

The evaluation method commonly used to assess the reliability of a test is the test-retest method. This method consists of administering the same test twice in the same individuals separated in time. If the test is consistent, the correlation between the test and retest will be high. However, this method supports stability in the evaluation and therefore seems inappropriate in the case of our knowledge questionnaire. Indeed, knowledge can fluctuate through time. Therefore, a method of assessing reliability that does not involve a double test is necessary in the case of our study. The internal consistency was notably estimated by the Cronbach α coefficient. It is considered good when the Cronbach α value is above .70 [72]. The health knowledge questionnaire was pretested on a population of 68 undergraduate students in psychology, which enabled us to confirm a standardized distribution of results (mean 20, median 20 [50% of correct answers], SD 5.3). The Cronbach α values were .72 for the blood pressure knowledge scale and .76 for the blood oxygenation knowledge scale, which can be considered good for both scales.

### User Statistics: Participant Demographics, IT Experience, and Medical Device Experience

Among the 149 participants (Table 2), we decided to retain only those who successfully used the medical devices in order not to bias efficiency results (ie, 137 for the blood pressure monitor and 147 for the pulse oximeter). Indeed, if a user handles the device for a very long time before definitively dropping it, it is not possible to take into account these measures of handling time (efficiency) because this will not correspond to the measurement criteria of this variable. We began by analyzing the impacts of user characteristics (eg, demographics, IT experience, and medical device experience) on usability. We then analyzed the correlation between the participant’s health knowledge and usability to test our hypotheses (H1, effectiveness; H2, efficiency; and H3, satisfaction) as follows: (1) for the blood pressure monitor, (2) for the pulse oximeter, and (3) the cluster analyses.

Age and level of education had no impact on usability results owing to the similar profiles of students (except gender) for effectiveness (*U*=1686.5, *P*=.02) and satisfaction (*U*=2756, *P*=.01) in the case of the pulse oximeter. The Kruskal-Wallis test revealed no significant difference between IT experience and usability results (effectiveness: χ²_2,136_=0.5, *P*=.77; efficiency: χ²_2,136_=0.6, *P*=.73; satisfaction: χ²_2,136_=0.2*, P*=.88) for the blood pressure monitor. Additionally, for the pulse oximeter, no significant difference was observed between IT experience and usability results (effectiveness: χ²_2,146_=1.8, *P*=.40; efficiency: χ²_2,146_=1.7, *P*=.43). However, participants who had already used a pulse oximeter were significantly more satisfied because they had a better SUS score (satisfaction: χ²_2,146_=6.036; *P*=.049). Details of these measures are available in Multimedia Appendix 3.

**Table 2:** Sociodemographic characteristics and experience (information technology and medical device) of the study cohort.

Variable			Value (N=149), n (%) or mean (SD)
**Sociodemographic characteristics**			
	Age (years)		20.72 (1.65)
	**Gender**		
		Male	37 (24.8%)
		Female	112 (75.2%)
	**Education level**		
		First year	2 (1.3%)
		Second year	88 (59.1%)
		Third year	59 (39.6%)
	**Information technology experience**		
		High	59 (43.1%)
		Medium	47 (34.3%)
		Low	31 (22.6%)
	**Comfort with computer use**		
		High	88 (59.0%)
		Medium	60 (40.3%)
		Low	1 (0.7%)
	**Frequency of computer use**		
		Every day (very often)	103 (69.1%)
		Several times a week (often)	39 (26.2%)
		Once in a while (seldom)	6 (4.0%)
		Never	0 (0%)
	**Comfort with cellphone use**		
		High	120 (80.5%)
		Medium	29 (19.5%)
		Low	0 (0%)
	**Frequency of cell phone use**		
		Every day (very often)	125 (83.9%)
		Several times a week (often)	19 (12.8%)
		Once in a while (seldom)	4 (2.7%)
		Never	0 (0%)
	**Connected device comfort**		
		High	10 (6.7%)
		Medium	31 (20.8%)
		Low	108 (72.5%)
	**Frequency of connected device use**		
		Every day (very often)	3 (2.0%)
		Several times a week (often)	4 (2.7%)
		Once in a while (seldom)	46 (30.9%)
		Never	95 (63.8%)
**Experience with the use of medical devices**			
	**Blood pressure monitor**		
		Yes	86 (57.7%)
		No	63 (42.3%)
	**Pulse oximeter**		
		Yes	20 (13.4%)
		No	129 (86.6%)

### Evaluation Outcomes

#### Blood Pressure Monitor

##### Usability Testing

One hundred and thirty-seven users were able to use the device successfully, that is, to obtain a measure (Table 3). Twelve participants gave up owing to the complexity of using the device. This result indicates a lack of effectiveness. For the rest of the sample, users made a mean of 0.77 errors (SD 1.49). Users manipulated the device for a mean of 260.91 seconds (SD 107.12). The mean SUS score was 72.4 (SD 21.07), which is “satisfactory” [67]. Regarding the descriptive results, this device appeared not to be at all user friendly for a population that has had no previous training. In addition, the data correctly recorded were extremely poor. Only 12 (9%) participants were able to correctly record their blood pressure.

**Table 3:** Results of the usability measurements of the blood pressure monitor and Spearman correlation between usability and prior health knowledge (N=137).

Measurement	Value		Correlation with health knowledge (*r*)	*P* value (unilateral)
	Mean (SD)	Range (minimum-maximum)		
Effectiveness (errors)	0.77 (1.49)	0-8	−0.191	.03
Efficiency (seconds)	260.91 (107.12)	90.5-681	−0.104	.23
Satisfaction (SUS^a^ score)	72.4 (21.07)	27.5-100	0.146	.09
Data recorded (rate of correct response)	0.099 (0.28)	0-1	0.302	<.001

^a^SUS: system usability scale.

##### Correlation of Usability With User Health Knowledge

Participants scored a mean of 10.16 out of 20 (SD 2.95, range 1-16), with 50.8% (n=69) correct answers. Spearman correlation between blood pressure knowledge and usability metrics showed low and negative but significant correlations between the number of errors and participant knowledge (*r*=−0.191, *P*=.03). Participants with a high level of health knowledge made fewer errors. The results were however not significant for efficiency (*r*=−0.104, *P*=.22) and satisfaction (*r*=0.146, *P*=.08). The majority of participants (n=12) misrecorded their data. We observed that only participants with a high level of health knowledge were able to record their blood pressure correctly (*r*=.302, *P*<.001). This is a medium, positive, and significant correlation that illustrates the limitations of this medical device. Although most participants knew how to manipulate the blood pressure monitor, they struggled to read the results.

#### Pulse Oximeter

##### Usability Testing

One hundred and forty-seven participants were able to use the pulse oximeter successfully (Table 4). Only two participants gave up because they failed to use the device properly, and their results were excluded from the analysis. The oximeter therefore appears to be easier to use than the blood pressure monitor, which was abandoned by 12 participants, but participants made more errors (mean 0.99, SD 0.92). The mean SUS score was 71.52 (SD 17.29), which is “satisfactory” [67]. In addition, data readings were quite good. On average, 64.6% (n=95) of the participants were able to record their oxygen levels correctly.

**Table 4:** Results of the usability measurements of the pulse oximeter and Spearman correlation between usability and prior health knowledge (N=147).

Measurement	Value		Correlation with health knowledge (*r*)		*P* value (unilateral)	
	Mean (SD)	Range (minimum-maximum)				
Effectiveness (errors)	0.99 (0.92)	0-6	−0.263		.001	
Efficiency (seconds)	158.42 (75.75)	24.8-458.8	−0.062		.45	
Satisfaction (SUS^a^ score)	71.52 (17.29)	15-100	0.195		.02	
Data recorded (rate of correct response)	0.65 (0.48)	0-1	0.018		.82	

^a^SUS: system usability scale.

##### Correlation of Usability With User Health Knowledge

For health knowledge of blood oxygenation, participants scored a mean of 5.28 out of 20 (SD 2.77, range 0-15), corresponding to 26.3% correct answers. Spearman correlation between blood pressure knowledge and ISO usability metrics showed similar results for the pulse oximeter and the blood pressure monitor. There was a low, negative, and significant correlation between the number of errors and participant knowledge (*r*=−0.263, *P*=.001). We may deduce that participants with better health knowledge make fewer mistakes and are therefore more efficient in handling the device. Significance for satisfaction and participant knowledge was also observed (*r*=0.195, *P*=.02). However, there was no correlation when reading the results (*r*=0.018, *P*=.82).

#### Cluster Analysis

In view of the poor correlations, we considered the possibility of a threshold effect. For this purpose, we created clusters (*k*-means), separating participants into three assignment groups according to their level of prior health knowledge. We then looked at the three knowledge groups (low, medium, and high) in terms of their *effectiveness, efficiency*, and *satisfaction* in relation to the blood pressure monitor (Table 5) and the pulse oximeter (Table 6).

**Table 5:** Prior health knowledge measurements by cluster groups according to usability metrics for the blood pressure monitor using the Kruskal-Wallis test (N=137).

Measurement	Low group (N=26; overall mean 8.73/40, SD 2.51)		Medium group (N=66; overall mean 14.47/40, SD 1.72)		High group (N=45; overall mean 20.93/40, SD 2.9)		Kruskal-Wallis test	
	Mean (SD)	Range	Mean (SD)	Range	Mean (SD)	Range	χ^2^ (*2,136*)	*P*
Effectiveness (errors)	92.00 (1.44)	0-7	0.86 (1.62)	0-8	0.56 (1.01)	0-4	2.5	.29
Efficiency (seconds)	286.42 (110.16)	112.1-534.3	257.53 (119.41)	90.5-681.04	251.14 (83.72)	127.5-494.2	2.9	.23
Satisfaction (SUS score)	70.38 (16.46)	27.5-100	72.39 (16.46)	27.5-97.5	73.82 (16.44)	40-100	1.0	.60
Health data read	0.00 (0.00)	0-0	0.05 (0.21)	0-1	0.20 (0.41)	0-1	3.5	.18

**Table 6:** Prior health knowledge measurements by cluster groups according to usability dimensions for the pulse oximeter using the Kruskal-Wallis test (N=147).

Measurement	Low group (N=44; overall mean 9.77/40, SD 2.76)		Medium group (N=29; overall mean 14.1/40, SD 0.77)		High group (N=74; overall mean 19.31/40, SD 3.28)		Kruskal-Wallis test	
	Mean (SD)	Range	Mean (SD)	Range	Mean (SD)	Range	χ^2^ (*2,146*)	*P*
Effectiveness (errors)	1.34 (0.88)	0-4	0.69 (0.6)	0-7	0.92 (1.04)	0-6	10.9	.004
Efficiency (seconds)	168.38 (76.98)	48.2-385.4	163.68 (77.47)	71.4-452.5	150.44 (74.49)	458.8-7	2.0	.37
Satisfaction (SUS score)	69.86 (16.38)	32-95	71.45 (23.23)	15-98	72.73 (15.16)	30-100	7.5	.02
Health data read	0.89 (0.32)	0-1	0.86 (0.35)	0-1	0.91 (0.29)	0-1	0.03	.98

Concerning effectiveness, the Kruskal-Wallis test revealed a significant difference between the number of pulse oximeter handling errors (effectiveness) and cluster groups (χ²_2,146_=10.9, *P*=.004). The low group (mean 1.3, SD 0.88) had significantly more errors than the other two groups. We performed the same analysis with efficiency, taking into account time and satisfaction using SUS measures. Efficiency did not reveal any significant difference (χ*²*_2,146_=2.0, *P*=.37). However, this threshold effect was found for satisfaction based on the SUS score (χ²_2,146_=7.5, *P*=.02), probably because of the number of errors made for the pulse oximeter. These results were not transferable to the blood pressure monitor.

## Discussion

### Main Contributions

The objective of this study was to explore how prior health knowledge, seen as part of the health literacy level [55,60,73], could impact the usability results (effectiveness, efficiency, and satisfaction) of HMDs. The findings support the central hypothesis of this study, namely that *better health knowledge leads to better usability*. Participants with good knowledge were more effective than those without good knowledge. More precisely, participants with knowledge of how blood pressure works in the human body made significantly (*P*=.03) fewer handling errors when using the blood pressure monitor. Having a basic understanding of how the body works, such as blood pressure, would help to better understand how the blood pressure monitor works and, for instance, prevent posture errors. This was the case for both devices, but a threshold effect was visible in the case of the oximeter. User tests also indicated that the blood pressure monitor was more difficult to use than the oximeter. Reading the result of a measurement seems to be intuitive in the case of the oximeter, but not in the case of the blood pressure monitor. The majority of participants were unable to read their blood pressure, indicating that they were unable to interpret it. Thus, participants can use the device correctly, but they need help to understand and interpret their physiological data. Understanding and interpreting data would require more knowledge.

**Figure 3:**
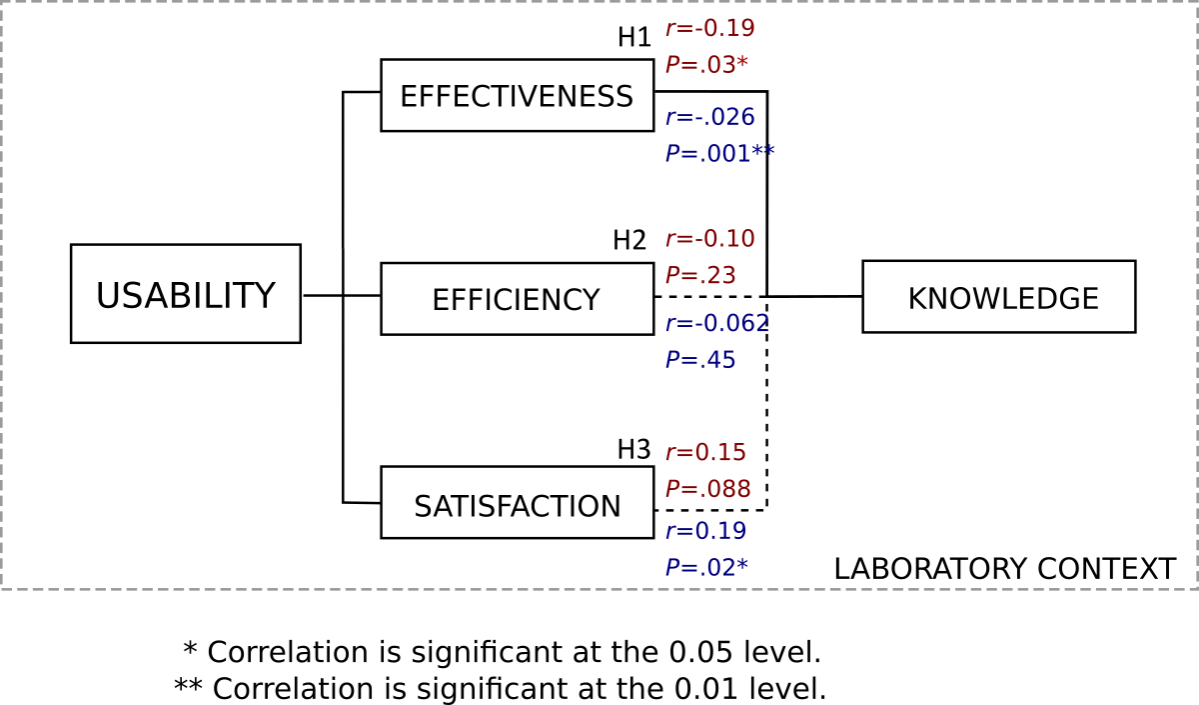
Synthesis of the results of the influence of prior health knowledge on usability results (ISO 9241-11) [15]. Results shown in red correspond to the blood pressure monitor, and those in blue correspond to the pulse oximeter.

In light of the above observations, our results appear to validate our hypothesis (Figure 3). Knowledge has an impact on *effectiveness* (H1) and partially on *satisfaction* (H3). However, the second hypothesis (H2) concerning the link between health knowledge and the handling time (efficiency) of these two devices could not be validated because no significant link could be observed.

**Figure 4:**
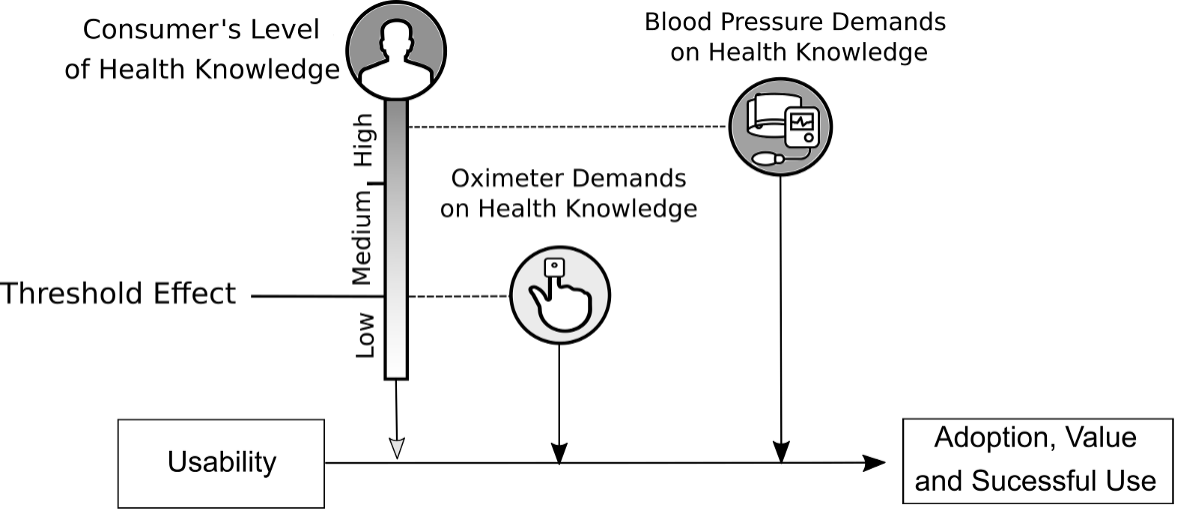
Adaptation of the Monkman and Kushniruk model [52] to the use of the blood pressure monitor and pulse oximeter.

These results may be explained by adapting the model from Monkman and Kushniruk [53] by switching the literacy level to the subject’s level of knowledge. The oximeter requires a lower level of knowledge on the part of the subject. As for the blood pressure monitor, it is worn on the wrist, which contradicts a widespread belief that blood pressure can only be monitored via the arm. The knowledge related to this device thus demands a greater effort to understand the operation of a blood pressure monitor and involves deeper knowledge. According to the Monkman and Kushniruk model, if a device’s “demands on eHealth literacy” exceed “consumers’ levels of eHealth literacy,” the adoption of the device is compromised. In this framework, the limit of understanding the pulse oximeter is likely to be located between the *low* group and the *medium* group, which explains the threshold effect (Figure 4). This interpretation is also supported by Paasche-Orlow and Wolf [57], who previously observed a threshold effect between health literacy and health outcomes. In contrast, the blood pressure monitor, unlike the pulse oximeter, requires a high level of knowledge in its use and in the reading of its results.

According to the Monkman and Kushniruk model [52], *adoption*, *value*, and *successful use* are related to both the usability and utility fields. However, one of the limitations of our study is that we performed this experiment with a young healthy population that had little interest in using HMDs. As a result, motivation may have affected our outcomes. For O’Brien and Toms [34], usability was linked to the engagement experience. A lack of participant motivation could give lower measures of system usability. In addition, only the performance of participants who successfully collected their health data with the devices was analyzed. If the participants who failed to collect their data had been included, the error count measures would certainly have increased and the SUS score would have decreased.

Another limitation of our study is that the health knowledge questionnaire was designed for the sort of distribution of knowledge that is to be found among a population of university students. However, in the overall population, the distribution is likely much wider than in a university population. This sample is not representative of the national population. For instance, this sample does not include individuals with language, reading, and writing difficulties. This poorly understood link between prior health knowledge and usability could be further explored in the future and especially among populations that are more representative of end users, such as populations that are older and have pathologies. Finally, the choice of equipment was made by health professionals who were accustomed to handling medical equipment. They judged for themselves the simplicity of the equipment, even though it was apparently complex to use. This observation highlights the importance of testing this type of equipment among novice users, which promotes universal design [74], and raises questions about the legislation on European conformity. It is possible that some devices are simpler to use than those chosen in this study. We encourage studies on medical devices that are already on the market in order to investigate possible difficulties in use by the general public [75].

### Research Perspectives

This is the first study aimed at detecting the first usability problems outside the context of use, which adds even more complexity. Indeed, the patient will be in a postsurgical context, with pain, nausea, and stress, and will often need the help of family members to use the devices at home. This first study of the *Smart Angel* project serves as a basis for comparing usability measurements on other specific contexts manipulating the four components of usability (*users*, *task* and *goal*, *environment*, and *resources*). A first research perspective is employed to define how the context of ambulatory surgery impacts the usability results of the system. If a patient with a high level of health knowledge makes use errors with a device, the learning context including pain and/or stress may be the cause of this error and therefore a poor usability result.

A second research perspective focuses on the acceptability of the device among patients and medical staff. During the acceptability phase, researchers will focus on the impact of the implementation of the system on the patient pathway and the organization of the hospital. To this end, a hospital study is being carried out in an outpatient surgery population. There is a need to further increase our knowledge of ergonomics on the factors that influence acceptability in order to improve patient safety.

### Recommendations for the Design and Integration of HMDs

Three types of recommendations based on the results obtained can be suggested. First, the results of this study could be used in the hospital to improve patient monitoring involving medical devices and to avoid use errors. They could help determine which devices would be the most suitable for the individual’s profile in terms of health understanding [76]. This information on a patient’s health knowledge would assist physicians in deciding if they need to recommend a particular device to that patient. However, this would require further studies on a population more representative of the national population.

Second, we were able to observe from our results that specific knowledge of the human body (eg, heart function, blood pressure, and blood oxygenation) made it possible to reduce the number of use errors, even though the individual had no experience with medical devices. Therefore, therapeutic education based on human body functioning, diseases, how to care for devices, and how devices work to meet the patient’s needs would be the key to making HMDs more usable [55]. Health professionals could provide anatomical and physiological explanations adapted to the HMDs chosen in terms of body functioning among patients, which will help in taking measurements correctly and preserving patient safety.

Third, we recommend that designers pay attention to the terms chosen. It is important to have a rigorous methodology on usability design and to follow the guidelines relating to the use of clear simple language in health care communication [77]. Just like medical professionals, designers can add playful information about the functioning of the human body to the device instructions.

### Conclusion

Our study has two main contributions. First, a scientific interest to provide theoretical knowledge about the factors influencing usability. Indeed, our findings indicate that prior knowledge influences the effectiveness of HMDs.

Second, our study has an applied interest to help designers and medical staff target the importance of providing specific knowledge of the subject to help patients understand how the device works. The results show that it is possible for HMDs to be well adapted to a low literacy level among patients. This was the case, for example, with the oximeter used in this study. Participants were not familiar with this device, and yet, they were able to use it and read their health results.

It should be noted that the study had some limitations. Our sample was restricted to younger adults with high levels of education and adequate health literacy. Clearly, usability needs to be evaluated in larger and more diverse user groups.

## Appendix

Multimedia Appendix 1 The French health knowledge questionnaire.

Multimedia Appendix 2 English translation of the prior health knowledge questionnaire.

Multimedia Appendix 3 Details of user characteristics based on usability measurements for the blood pressure monitor and the pulse oximeter.

## Abbreviations

CE: European conformity
HMD: home medical device
ICC: intraclass correlation coefficient
IT: information technology
SUS: system usability scale
