# Supplementary material for: Effect of Prior Health Knowledge on the Usability of Two Home Medical Devices: Usability Study
Source: JMIR Mhealth Uhealth. 2020 Sep 21;8(9):e17983. doi: 10.2196/17983 (PMC7536595; doi:10.2196/17983)
Supplement: Multimedia Appendix 1 [file mhealth_v8i9e17983_app1.docx]

Table S1 : French questionnaire on prior health knowledge (40 items).

|  | Vrai | Faux | Ne sais pas |
| --- | --- | --- | --- |
| 1.     Le cycle cardiaque |  |  |  |
| a.      Le cycle cardiaque comprend une contraction suivie d’un relâchement |  |  |  |
| b.     Le cycle cardiaque comprend une inspiration suivie d’une expiration |  |  |  |
| c.     La contraction cardiaque est appelée diastole |  |  |  |
| d.      Le cœur fonctionne comme une pompe qui propulse le sang vers les tissus |  |  |  |
|  |  |  |  |
| 2.     Pression artérielle |  |  |  |
| a.      La pression artérielle peut être définie comme la force exercée par le sang sur la paroi des artères. |  |  |  |
| b.     La pression artérielle peut être définie comme le volume de sang éjecté par le cœur. |  |  |  |
| c.      La pression artérielle peut être définie comme le nombre de battements du cœur par minute. |  |  |  |
| d.     La pression artérielle est synonyme de la tension artérielle. |  |  |  |
|  |  |  |  |
| 3.     Les valeurs de référence ou valeurs « normales » |  |  |  |
| a.      Les deux chiffres obtenus lors de la mesure de la tension correspondent à la valeur de référence suivie de la valeur obtenue par le patient |  |  |  |
| b.     Les valeurs de référence chez les sujets jeunes sont 140 / 90 mmHg |  |  |  |
| c.      Avec l’âge, la tension artérielle baisse. |  |  |  |
| d.    Plus l’artère est rigide et plus les deux chiffres sont élevés. |  |  |  |
|  |  |  |  |
| 4.     Variation de tension |  |  |  |
| a.      On parle d’hypertension quand les deux chiffres sont supérieurs aux valeurs de référence |  |  |  |
| b.      Le cerveau est le seul organe épargné dans le cas d’une hypertension de longue durée. |  |  |  |
| c.     Une hémorragie peut entrainer une hypotension |  |  |  |
| d.      L’hypotension peut entrainer la perte de connaissance |  |  |  |
|  |  |  |  |
| 5.     L’utilisation du tensiomètre |  |  |  |
| a.      Le tensiomètre se met généralement au bras |  |  |  |
| b.     Le tensiomètre nécessite l’application d’un gel |  |  |  |
| c.      L’utilisation du tensiomètre mesure l’activité électrique des artères |  |  |  |
| d.      Seul un personnel médical peut utiliser un tensiomètre. |  |  |  |
|  |  |  |  |
| 9 questions faciles, 9 questions moyennes, 2 questions difficiles / 20 questions totales |  |  |  |
|  |  |  |  |
| Questions saturometre : |  |  |  |
| 6.     Le rôle des poumons : |  |  |  |
| a.      L’oxygène traverse les parois des alvéoles dans les poumons |  |  |  |
| b.     Le temps de contact entre les alvéoles et l’hémoglobine permet la saturation des hématies en oxygène. |  |  |  |
| c.      C’est le cœur qui permet la répartition de l’oxygène dans les tissus (organes, muscles…) |  |  |  |
| d.      L’oxygène est transporté dans le sang grâce aux globules blancs |  |  |  |
| 7.     Transport des gaz respiratoires dans le corps |  |  |  |
| a.     Le dioxyde de carbone est éliminé par les reins |  |  |  |
| b.      Le sang riche en dioxyde de carbone et le sang riche en oxygène ne se rencontrent pas. |  |  |  |
| c.     Le sang circule en sens unique dans un système clos. |  |  |  |
| d.      Le dioxyde de carbone est produit par les tissus (organes, muscles…). |  |  |  |
|  |  |  |  |
| 8.  Saturomètre |  |  |  |
| a.     Le saturomètre mesure le taux d’oxygène dans le sang |  |  |  |
| b.     Permet de repérer les carences en oxygène dans les tissues |  |  |  |
| c.      Permet de repérer les anémies |  |  |  |
| d.     L’utilisation du saturomètre nécessite une prise de sang. |  |  |  |
|  |  |  |  |
| 9.  Le taux d’oxygène |  |  |  |
| a.      Le taux d’oxygène dans le sang a tendance à baisser avec l’âge |  |  |  |
| b.     Le taux d’oxygène dans le sang diffère selon le sexe |  |  |  |
| c.     Les fumeurs ont tendance à avoir une saturation en oxygène plus importante que celle des non-fumeurs. |  |  |  |
| d.      Une valeur inférieure de saturation en oxygène à 60% est signe d’hypoxémie. |  |  |  |
|  |  |  |  |
| 10. l’utilisation du saturomètre |  |  |  |
| a.     Le saturomètre peut être positionné sur le lobe de l’oreille |  |  |  |
| b.      L’utilisation du saturomètre nécessite l’application d’un gel |  |  |  |
| c.     Seul un personnel médical peut utiliser un saturomètre. |  |  |  |
| d.      Le vernis à ongle peut fausser les valeurs données par le saturomètre |  |  |  |
