# Supplementary material for: Effect of Prior Health Knowledge on the Usability of Two Home Medical Devices: Usability Study
Source: JMIR Mhealth Uhealth. 2020 Sep 21;8(9):e17983. doi: 10.2196/17983 (PMC7536595; doi:10.2196/17983)
Supplement: Multimedia Appendix 2 [file mhealth_v8i9e17983_app2.docx]

Table S2. English translation of the prior health knowledge questionnaire.

| Blood pressure questions : | True | False | Don’t know |
| --- | --- | --- | --- |
| 1. The heart cycle |  |  |  |
| a. The cardiac cycle consists of a contraction followed by a relaxation. |  |  |  |
| b. The cardiac cycle consists of one inhalation followed by one exhalation. |  |  |  |
| c. The cardiac contraction is called diastole. |  |  |  |
| d. The heart acts as a pump that propels blood to the tissues and tissues of the body. |  |  |  |
|  |  |  |  |
| 2.     Blood pressure |  |  |  |
| a.      Blood pressure can be defined as the force exerted by the blood on the walls of the arteries. |  |  |  |
| b.     Blood pressure can be defined as the volume of blood ejected by the heart. |  |  |  |
| c.      Blood pressure can be defined as the number of heart beats per minute. |  |  |  |
| d.     Blood pressure is synonymous with “arterial pressure”. |  |  |  |
|  |  |  |  |
| 3.     The reference or "normal" values |  |  |  |
| a.      The two values obtained from the blood pressure measurement are the reference value followed by the value obtained by the patient. |  |  |  |
| b.     The reference values in young subjects are 140 / 90 mmHg |  |  |  |
| c.      With age, blood pressure drops. |  |  |  |
| d.    The more inflexible the artery, the higher the two numbers. |  |  |  |
|  |  |  |  |
| 4.     Blood pressure variation |  |  |  |
| a.      Hypertension is defined as when both numbers are above the reference values. |  |  |  |
| b.      The brain is the only organ spared in long-term hypertension |  |  |  |
| c.     A hemorrhage can lead to hypotension. |  |  |  |
| d.      Hypotension can lead to unconsciousness. |  |  |  |
|  |  |  |  |
| 5.     The use of the blood pressure monitor |  |  |  |
| a.      The monitor is usually placed on the arm |  |  |  |
| b.     The blood pressure monitor requires the application of a gel. |  |  |  |
| c.      The use of the blood pressure monitor measures the electrical activity of the arteries. |  |  |  |
| d.      Only medical personnel may use a blood pressure monitor. |  |  |  |
|  |  |  |  |
| Pulse Oximeter questions: |  |  |  |
| 6.     The role of the lungs: |  |  |  |
| a.      Oxygen passes through the walls of the alveoli in the lungs |  |  |  |
| b.     The contact time between the alveoli and the haemoglobin allows the oxygen saturation of the red blood cells. |  |  |  |
| c.      It is the heart that allows the distribution of oxygen in the tissues (organs, muscles...) |  |  |  |
| d.      Oxygen is transported in the blood by white blood cell |  |  |  |
|  |  |  |  |
| 7.     Transport of respiratory gases in the body |  |  |  |
| a.     Carbon dioxide is eliminated by the kidneys. |  |  |  |
| b.      Carbon dioxide-rich blood and oxygen-rich blood do not meet. |  |  |  |
| c.     Blood flows in a closed system in one direction only. |  |  |  |
| d.      Carbon dioxide is produced by tissues (organs, muscles, etc.). |  |  |  |
|  |  |  |  |
| 8.  Pulse oximeter |  |  |  |
| a.     The pulse oximeter measures the level of oxygen in the blood. |  |  |  |
| b.     The pulse oximeter identifies oxygen deficiencies in tissues. |  |  |  |
| c.      The pulse oximeter identifies anemias. |  |  |  |
| d.     The use of the pulse oximeter requires a blood test. |  |  |  |
|  |  |  |  |
| 9.  Oxygen level |  |  |  |
| a.      Oxygen levels in the blood tend to decrease with age. |  |  |  |
| b.     The level of oxygen in the blood differs according to gender |  |  |  |
| c.     Smokers tend to have higher oxygen saturation than non-smokers. |  |  |  |
| d.      A lower oxygen saturation value of 60% is a sign of hypoxemia. |  |  |  |
|  |  |  |  |
| 10. the use of the pulse oximeter |  |  |  |
| a.     The pulse oximeter can be positioned on the earlobe. |  |  |  |
| b.      The use of the hydrometer requires the application of a gel |  |  |  |
| c.     Only medical personnel may use a pulse oximeter. |  |  |  |
| d.      Nail polish can distort the values given by the pulse oximeter. |  |  |  |
