# Supplementary material for: Effect of Prior Health Knowledge on the Usability of Two Home Medical Devices: Usability Study
Source: JMIR Mhealth Uhealth. 2020 Sep 21;8(9):e17983. doi: 10.2196/17983 (PMC7536595; doi:10.2196/17983)
Supplement: Multimedia Appendix 3 [file mhealth_v8i9e17983_app3.docx]

Table 7: details of user characteristics based on usability measurements for the blood pressure monitor.

| **Variable (N=137)** | | **Effectiveness**  **(errors)** | **Efficiency (seconds)** | **Satisfaction**  **(SUS)** |
| --- | --- | --- | --- | --- |
| **Sociodemographic Characteristics** | | | | |
| Age in years (Mean (SD) ;) | **Mean (SD)**  N = 149 | .77 (1.41) | 260.91 (107.12) | 72.48 (16.23) |
|  | Spearman unilateral correlation | *r* = -.054,  *P* = .53 | *r* = - .065, *P* = .45 | *r* = .002,  *P* = .97 |
| Gender | Male (n = 37)  Female (n = 100) | 0.7 (1.56)  0.8 (1.36) | 266.11 (106.64)  258.99 (109.72) | 73.09 (16.13)  72.25 (16.12) |
|  | Mann-Whitney | U = 1646  *P* = .26 | U = 1892  *P* = .84 | U = 1899  *P* = .81 |
| Education Level | 1st year (n = 2)  2nd year (n = 80)  3rd year (n = 55) | 1. (0)   0.87 (1.55)  0.67 (1.21) | 156.14 (1.19)  270.41 (112.2)  250.63 (100.1) | 80 (10.61)  72.37 (16.89)  72.08 (15.56) |
|  | Kruskal-Wallis | χ²= 2.43  *P* = .49 | χ²= 3.16  *P* = .37 | χ²= 1.74  *P* = .63 |
| **Information Technology (IT) Experience** | High (n = 47; 29.51 (2.46))  Medium (n =59; 23.63 (1.74))  Low (n = 31; 24.39 (4.83) | 0.85 (1.61)  0.59 (0.97)   1. (1.75) | 266.3 (122.3)  258.45 (112.68)  257.43 (66.83) | 71.69 (16.78)  72.63 (15.66)  73.39 (16.93) |
|  | Kruskal-Wallis | χ²= 0.51  *P* = .77 | χ²= 0.64  *P* = .73 | χ²= 0.24  *P* = .88 |
| **Experience in the use of medical device** | | | | |
| Blood pressure monitor | Yes (n =61)  No (n = 76) | 0.66 (1.26)  0.87 (1.52) | 250.81 (100.4)  269.02 (112.22) | 72.54 (16.03)  72.43 (16.5) |
|  | Mann-Whitney | U = 2490.5,  *P* = .39 | U = 2529,  *P* = .36 | U = 2364,  *P* = .84 |
| Pulse oximeter | Yes (n = 19)  No (n = 118) | 0.68 (0.75)  0.79 (1.49) | 264.61 (96.73)  260.32 (109.07) | 75.79 (15.34)  71.94 (16.37) |
|  | Mann-Whitney | U = 1247.5,  *P* = .37 | U = 1142, *P* = .89 | U = 1286,  *P* = .3 |

Table 8: details of user characteristics based on usability measurements for the pulse oximeter

| **Variable (N=147)** | | **Effectiveness**  **(errors)** | **Efficiency (seconds)** | **Satisfaction**  **(SUS)** |
| --- | --- | --- | --- | --- |
| **Sociodemographic Characteristics** | | | | |
| Age in years (Mean (SD) ;) | **Mean (SD)**  N = 147 | 0.99 (0.921) | 158.42 (75.75) | 75.75 (17.29) |
|  | Spearman unilateral correlation | *r* = .068,  *P* = .42 | *r* = - .012, *P* = .89 | *r* = -.027,  *P* = .75 |
| Gender | Male (n = 41)  Female (n = 106) | 0.73 (0.77)  1.08 (0.96) | 139.7 (62.5)  165.67 (79.38) | 77.68 (14.65)  69.27 (17.72) |
|  | Mann-Whitney | U = 1686.5  *P* = .02* | U = 1768  *P* = .08 | U = 2756  *P* = .012* |
| Education Level | 1st year (n = 2)  2nd year (n = 86)  3rd year (n = 59) | 1. (0)   1 (0.98)  .93 (0.83) | 144.2 (116.75)  149 (68.72)  172.22 (84) | 67 (7.071)  73.62 (15.8)  68.47 (19.2) |
|  | Kruskal-Wallis | χ²= 4.37  *P* = .22 | χ²= 3.07  *P* = .38 | χ²= 4.61  *P* = .2 |
| **Information Technology (IT) Experience** | High (n = 46; 29.52 (2.49))  Medium (n =58; 23.59 (1.73))  Low (n = 31; 18.1 (2.72) | 0.89 (0.8)  .95 (1.02)   - 1. (0.97) | 164.97 (85.83)  157.87 (71.2)  174.28 (69.81) | 76.04 (17.2)  71.02 (16.02)  69.1 (16.01) |
|  | Kruskal-Wallis | χ²= 1.83  *P* = .4 | χ²= 1.68  *P* = .43 | χ²= 6.036  *P* = .049* |
| **Experience in the use of medical device** | | | | |
| Blood pressure monitor | Yes (n =62)  No (n = 85) | 0.97 (0.77)  1 (1.02) | 158.65 (86.93)  158.26 (66.98) | 71.29 (18.17)  71.86 (16.73) |
|  | Mann-Whitney | U = 2565.5,  *P* = .76 | U = 2779,  *P* = .57 | U = 2682,  *P* = .85 |
| Pulse oximeter | Yes (n = 19)  No (n = 128) | 0.95 (0.7)  0.99 (0.95) | 179.01 (57.31)  155.37 (77.84) | 77.53 (15.43)  70.74 (17.43) |
|  | Mann-Whitney | U = 1255.5,  *P* = .8 | U = 1526, *P* = .07 | U = 1501,  *P* = .09 |
